# Brain Microstructural Alteration and Its Implications for Postoperative Delirium in Elderly Surgical Patients: A Narrative Review

**DOI:** 10.1002/brb3.70872

**Published:** 2025-09-10

**Authors:** Yali Chen, Huanyu Luo, Jing Dong, Jun Zhang

**Affiliations:** ^1^ Department of Anesthesiology Fudan University Shanghai Cancer Center Shanghai China; ^2^ Department of Oncology, Shanghai Medical College Fudan University Shanghai China

**Keywords:** brain microstructure, brain reserve, elderly patients, neuroimage, postoperative delirium

## Abstract

**Purpose:**

Postoperative delirium (POD) remains poorly understood in terms of predictors and underlying mechanisms. This review summarized emerging evidence on the association between brain microstructural alterations and POD.

**Method:**

This is a narrative review, describing the microstructural changes in aging brain, microstructural MRI findings, relationship among microstructural alterations, cognitive reserve and POD, and potential interventions targeting microstructure.

**Finding:**

Brain microstructural alterations may predispose elderly patients to POD, with variability in microstructural alterations in gray/white matter organization potentially determining the fate of brain aging and performance of cognitive reserve. Advanced neuroimaging techniques could play a critical role in understanding the microstructural changes, enabling better prediction of POD and elucidation of its pathogenesis. Enhancing brain resilience to mitigate the adverse effects of surgical trauma may prove vital in preventing POD.

**Conclusion:**

Pre‐existing brain microstructural alterations may be associated with subsequent development of POD, interventions targeting brain microstructure may decrease POD risk.

## Graphical abstract

Aging brains have differential microstructural alterations which may determine who is susceptible to POD in elderly surgical patients. Specific structural biomarkers detected by advanced MRI techniques may help to identify those at high‐risk of POD, while preoperative cognitive interventions associated with microstructural improvements may decrease the risk of POD.

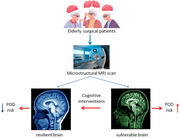

**Abbreviations**
AD: Alzheimer's disease
Aβ: amyloid‐beta
BBB: blood–brain barrier
CSF: cerebral‐spinal fluid
DKI: diffusion kurtosis imaging
dMRI: diffusion MRI
DTI: diffusion tensor imaging
EEG: electroencephalographic
FA: fractional anisotropy
MCI: mild cognitive impairment
MD: mean diffusivity
MRI: magnetic resonance imaging
PND: postoperative neurocognitive disorders
POD: postoperative delirium
SVD: small vessel disease
T2DM: type 2 diabetes
TBI: traumatic brain injury
tDCS: transcranial direct current
TMS: transcranial magnetic stimulation

## 1 Introduction

Postoperative delirium (POD) is one of neurological complications following major surgery especially in elderly patients, characterized by acute‐onset attention deficits, impaired consciousness, and disruptions in memory, language, visuospatial function, or perception (Inouye et al. 2014; Jin et al. 2020). POD is associated with prolonged hospitalization, long‐term cognitive decline, and mortality (Bilotta et al. 2010), posing a significant public health challenge. Despite its clinical importance, the neural mechanism underlying POD is incompletely understood, hindering its effective therapies in clinical practice (Duning et al. 2021). The neuroinflammation, oxidative/endoplasmic reticulum stress, and mitochondrial dysfunction have been regarded as key drivers of POD (Marshall et al. 2025). However, the underlying brain structural correlates of predisposition to POD remain largely unknown.

Currently several risk factors have been used for predicting POD, which can be categorized into four domains (Foley and Djaiani 2025): (1) old age; (2) pre‐existing systemic or neurological diseases (Marcucci et al. 2023); (3) pre‐existing cognitive impairments; (4) perioperative complications leading to brain damage. These risk factors are commonly correlated with neuropathology and brain microstructural alteration. For example, aged brain tends to amyloid‐beta (Aβ) accumulation and brain atrophy although most of elderly patients exhibit intact cognitive function preoperatively (Bugiani 2021). And preoperative comorbidities such as cardiopulmonary diseases could significantly affect brain structures (Spilling et al. 2021; Xiong et al. 2019; Tsiknia et al. 2023), leading to increased risk and burden of POD.

Therefore, preoperative brain microstructural alternations may represent a neural vulnerability, while surgery‐triggered pathophysiology may intensify this vulnerability, leading to development of POD. To improve our understanding of the pathogenesis of POD, here we summarize recent studies on the relationship between brain microstructural changes and POD, and provide potential predictors associated with POD and the implications for POD prevention.

## 2 Why Elderly Patients Are Prone to POD

Aging is known to potentiate inflammation due to immune cell senescence, increase vascular permeability due to blood–brain barrier (BBB) disruption, and decrease cerebral blood flow and neuroenergetic metabolism due to neurovascular uncoupling, which account for changes in brain structure (Graff et al. 2023; Hu et al. 2025). As a result, reduction in the neural cells number, neurogenesis, synaptic connections and brain volume, the myelin degradation, and the conduction velocity slowdown as well as imbalances of neuronal microenvironment homeostasis commonly develop in the aged brain (Zhou et al. 2024). Furthermore, systemic diseases, primary encephalopathies such as TDP‐43 proteinopathy, or genetic factors such as APOEε4 allele carrier may accelerate these processes, manifesting as brain macro‐ and microstructural alterations that precede cognitive decline. Intriguingly, a magnetic resonance imaging (MRI) study has revealed that microstructural—rather than macrostructural volumetric—changes correlate more strongly with neurobehavioral performance (Singh et al. 2024).

### 2.1 Microstructural Alterations in Aging Brain

Brain microstructure exhibits age‐related degenerative changes during normal aging. Advances in quantitative MRI sensitive to brain's myelin, iron, and free water (FW) content allow for a detectable in vivo investigation of aging‐related microstructural changes. Increased water in extracellular and intracellular space, decreased neurite density and complexity, have been observed in the cortical gray matter, hippocampus, and white matter tracts of elderly individuals (Reas, Hagler Jr., Andrews, et al. 2020). In a large‐scale cohort study, Taubert et al. (2020) confirmed age‐related brain atrophy, loss in white matter volume, myelin, and FW decreases in sensorimotor and subcortical areas (e.g., frontostriatal projections), highlighting the vulnerability of motor and executive networks in aging brain.

### 2.2 Aging‐Related Gray Matter Microstructural Alterations

Human Connectome Project‐aging cohort data (*n* = 707) demonstrate strong associations between age and gray matter microstructural alterations, including widespread cellular morphological changes, reduced cellular density, and increased extracellular volume and membrane permeability (Singh et al. 2024). Structurally, even in cognitively healthy ageing, volumes of hippocampal and anterior thalamic gray matter decrease with age (Frank et al. 2022). Specially, hippocampus, a critical region for memory and learning, displays age‐related microstructural deterioration in subfields dentate gyrus and CA3 region (Vinci‐Booher et al. 2023). And cortical plasticity in the aging individuals becomes increasingly important for structural reserve and functional compensation. For example, in nondemented older individuals with severe ischemic small vessel disease (SVD), the cortical plasticity compensatively increases when lower white matter integrity in parahippocampal regions and posterior corpus callosum (List et al. 2013). However, changes in macrostructure, such as cortical thickness, cannot completely inform about the underlying neuroplastic pathophysiology. In turn, cortical microstructural changes appear to precede macrostructural cortical atrophy in a more sensitive way. Further, cortical neurodegeneration and white matter hyperintensity are not two irrelevant processes. It is found in cognitively healthy elderly adults, lower initial cortical thickness has faster white matter progression, while faster white matter progression is associated with accelerated cortical thinning. This mutually reinforcing relationship become entangled before cognitive deficits are significant (Bernal et al. 2024). Therefore, microstructural measurements in gray matter may improve early risk identification of cognitive decline.

### 2.3 Aging‐Related White Matter Microstructural Alterations

Aging is also associated with widespread alterations in white matter. A multimodal MRI study has indicated in the absence of gross white matter atrophy, widespread but regional heterogeneous changes of white matter microstructure are associated with aging (Kelley et al. 2021). Specifically, age‐related differences in fiber density were prominent in fornix, bilateral anterior internal capsule, forceps minor, body of the corpus callosum, and corticospinal tract, while age differences in fiber cross section are largest in cingulum bundle and forceps minor. Furthermore, decreases in axon density and alterations in axonal structure are also found in aged brain. The axon diameter in the aging corpus callosum decreases more pronounced in the genu than splenium when using a diffusion MRI (dMRI)‐derived metric axon diameter index. Correspondingly, larger axon diameter index is associated with poorer cognitive performance (Fan et al. 2019).

### 2.4 Microstructural Alteration and Cognitive Functions

Gray/white matter microstructural integrity correlates with global cognition, executive function, and visuospatial, episodic, and logical memories. For women, hippocampal and corpus callosum microstructure mediates age‐related effects on verbal and visuospatial memories, respectively, whereas for men fiber microstructure (mainly fornix and corpus callosum) mediates executive function and visuospatial memory (Reas et al. 2021). It is found that deterioration in white matter microstructure driven by the presence of cardiovascular risk factors may mediate cognitive decline on the Montreal cognitive assessment in community‐dwelling older adults (Jolly et al. 2016). In elderly Chinese adults, both subjective cognitive decline and mild cognitive impairment (MCI) are significantly associated with cortical thinning or reduced gray matter volume in certain brain areas (Chen et al. 2024), while Ritchie et al. (2015) observed coupled changes in white matter microstructure and cognitive performance in elderly individuals, and the changes in microstructural integrity in medial temporal lobe subfields is also found associated with cognitive decline in aging individuals and those with MCI (Wearn et al. 2021). Therefore, maintaining brain microstructure integrity is essential for preserving cognitive function with aging (Lövdén et al. 2014).

These findings suggest that: (1) microstructural changes in elderly individuals can be used to predict cognitive decline; (2) subtle brain microstructural alterations can be captured by MRI in cognitively unimpaired individuals; (3) a structural basis of cognitive deterioration may be more susceptible to POD in elderly surgical patients.

## 3 Why Some of Them Experience POD

Pre‐existing comorbidities or neuropathology may play an important role in brain microstructural changes, while the surgical patients with these disorders are considered at high‐risk of POD.

### 3.1 Pre‐Existing Comorbidities and Microstructural Alterations

#### 3.1.1 Cardiopulmonary Diseases

These comorbidities could predispose elderly patients POD occurrence. Changes in brain structure and cognitive decline occur in chronic obstructive pulmonary disease and coronary artery disease. The poor respiratory function, higher blood pressure, and higher C‐reactive protein level are associated with white matter microstructural changes (Spilling et al. 2021). The cardiovascular diseases‐related inflammation, oxidative stress, neuroendocrine imbalances, and impaired cerebral perfusion can lead to white matter microstructural lesion and brain volume loss (Wang et al. 2025).

#### 3.1.2 Metabolic Disorders

The metabolic disorders are associated with an increased risk of POD in elderly patients undergoing noncardiac surgery (Feinkohl et al. 2023; hang et al. 2024; Zhao et al. 2024). The microstructural changes in white matter with an anterior–posterior pattern are found in individuals with metabolic syndrome when compared with age‐matched controls (Savolainen 2010). Similarly, the brain microstructural alterations are also detected in the patients with type 2 diabetes (T2DM) (Xiong et al. 2019). Specially, white matter microstructural changes in T2DM patients mainly locate in the corpus callosum, internal capsule, external capsule, corona radiata, posterior thalamic radiations, sagittal stratum, cingulate gyrus, fornix, superior longitudinal fasciculus, and uniform fasciculus when compared with healthy control (Gao et al. 2024). These evidences indicate a pathology of diabetic encephalopathy. Dislipidemia is another risk factor of POD (Zhao et al. 2024). Iriondo et al. investigated the relationship between plasma lipids and white matter microstructure, and found that levels of low‐density lipoprotein and triglyceride had a negative association with white matter microstructural integrity and a positive association with axonal degeneration in healthy adults (Iriondo et al. 2021).

#### 3.1.3 Sleep Disorders

Nearly half of the adults over 60 years of age experience sleep disorders, which has been associated with brain atrophy and dementia. The poor subjective sleep quality before surgery is independently associated with increased POD risk (Ou‐Yang et al. 2024). A recent study found that the poor sleep quality was associated with lower white matter restricted isotropic diffusion, neurite density, and higher amygdala FW (Tsiknia et al. 2023), suggesting a contribution of sleep disorders to brain microstructural damage in cognitively healthy older adults. And in the patients with obstructive sleep apnea syndrome, microstructural changes in white matter are associated with cognitive impairments (Ning et al. 2024). Surprisingly, untreated sleep apnea is reported not associated with increased incidence or severity of postoperative neurocognitive disorders (PND) or POD in older patients undergoing noncardiac surgery (Devinney et al. 2025).

#### 3.1.4 Economical and Environmental Factors

The socioeconomic status of surgical patients has emerged as a novel and important independent risk factor for POD (Arias et al. 2024; Arias et al. 2020). It is found that socioeconomic status is an important reserve variable, which modulates the aging brain's microstructure. The elderly adults with high socioeconomic status show lower age‐related white matter integrity declines in three frontal tracts: the right anterior corona radiata and bilateral white matter underlying the superior frontal gyri (Johnson et al. 2013). In contrast, negative neuroplasticity resulting from marital status (divorced, widowed, and separated), economic, or environmental impoverishment, commonly seen in elderly adults, can lead to detrimental changes in brain structure (i.e., lower entorhinal cortex thickness) and function (Lee et al. 2024; Fenton et al. 2024), which may exacerbate age‐related cognitive decline over time.

### 3.2 Brain Neuropathology and Microstructural Alterations

Accumulation of Aβ plaques and tau tangles are common neuropathological features in aged brain, which may lead to microstructural changes. These neuropathological changes precede the emergence of neurocognitive deficits. For example, hippocampal CA1 field and inferior temporal cortex display increasing densities of neurofibrillary tangles with age, whereas the superior frontal and occipital cortices are relatively spared (Giannakopoulos et al. 1994). The amyloid is correlated with widespread microstructural brain changes, conversely, microstructural abnormalities can predict cognitive decline regardless of amyloid (Reas, Hagler Jr., Kuperman, et al. 2020), suggesting microstructural changes are more sensitive than amyloid in predicting cognitive decline. The topographic difference in Aβ plaques and tau tangles suggest an age‐related heterogeneity in the regional brain vulnerability during normal brain aging. Further, microstructural changes are also sensitive to tau and amyloid accumulation in cognitively unimpaired individuals at the earliest stages of pre‐clinical Alzheimer's disease (AD) (Cacciaglia et al. 2024). For the same p‐tau/Aβ42 level in cerebral‐spinal fluid (CSF), those older individuals who have both greater cortical Aβ deposition and lower gray matter volume are more likely to suffer from neuropathological events. Actually, preoperative levels of these AD biomarkers in plasma or CSF are linked to POD (Liang et al. 2023; Geng et al. 2024).

### 3.3 Microstructural Spatiotemporal Changes and Brain Aging

Although microstructural changes commonly in aging brain, cognitive sequelae vary considerably. We speculate the spatiotemporal heterogeneity in microstructural changes may determine the fate of brain aging, as well as the development of POD. One study using the UK Biobank data exhibited that cortical thinning initially in the right hemisphere, followed by the temporal lobe in elderly people with accelerated brain aging; in contrast, initial cortical thinning predominantly in the left hemisphere and white matter microstructural degeneration occurring at more advanced stages in those with resilient brain aging (Lin et al. 2024). While microstructural integrity of the anterior mid‐cingulate cortex may be a useful brain resilience biomarker against POD in cognitively normal elderly patients undergoing elective surgery (Katsumi et al. 2022). Therefore, the specific location and severity of microstructural changes may determine who will finally develop into POD, even evolve into AD.

## 4 The Cognitive Reserve and Brain Microstructural Changes

### 4.1 Definition of Cognitive Reserve

Cognitive or brain reserve refers to the brain's ability to cope with damage or pathology, which has been a topic of interest in the studies related to age‐associated cognitive impairment and delirium. The idea behind cognitive reserve is that certain factors, such as education level, premorbid brain volume, and intellectual enrichment, may help individuals maintain cognitive function in the face of brain injury or aging as a form of “brain resilience.” For example, educational attainment has been identified as a key contributor to cognitive reserve, with higher education levels associated with better cognitive outcomes in older age (Mungas et al. 2021), while both lower educational level and preoperative mini‐mental status examination score are risk factors of POD.

### 4.2 Neural Basis of Cognitive Reserve

The neural basis of cognitive reserve may be associated with brain activation patterns. A significant correlation between change in brain activity and the cognitive score during a recognition memory task indicates that cognitive reserve may influence how the brain responds to cognitive challenges (Stern et al. 2003; Vockert et al. 2024). The higher cognitive reserve is related to larger brain volume and reduced activity during cognitive task in healthy elders (Solé‐Padullés et al. 2009), while larger volumes of hippocampus or hippocampal subfield may act as a protective neural reserve against stress (Koch et al. 2021), suggesting an acquired neural effect. Microstructural integrity may be responsible for the brain activation patterns, however, it is not clear whether changes in preoperative electroencephalographic (EEG) features or functional MRI‐derived metrics in the patients subsequently developing into POD (Pollak et al. 2024; Tanabe et al. 2020; Browndyke et al. 2021) indicate a specific change in brain activity associated with microstructural alternations.

### 4.3 Cognitive Reserve and Microstructural Changes

Several studies with different methodologies and populations have investigated the relationship between cognitive reserve and brain microstructural changes. Bendlin et al. (2010) studied the white matter microstructure in relation to age and neuropsychological function using diffusion tensor imaging (DTI), and found that white matter microstructure could provide unique information on brain aging beyond what is explained by white matter volume. And in individuals with traumatic brain injury (TBI), larger premorbid brain volume and higher education levels were found associated with decreased vulnerability to cognitive deficits (Mathias and Wheaton 2015). While among Aβ‐positive individuals, greater cognitive reserve relates to attenuated clinical progression in prodromal stages of AD (van Loenhoud et al. 2019). These findings highlight the importance of microstructural changes in cognitive reserve. However, an association of cognitive reserve and brain macrostructural alterations in elderly adult has not been established (Kalzendorf et al. 2020).

### 4.4 Cognitive Reserve and POD

Preoperative cognitive impairment is found significantly correlated with POD and PND (Knaak et al. 2020; Varpaei et al. 2024), while higher cognitive reserve, as measured by literacy and cognitive activity participation, is associated with reduced incidence and severity of POD in older surgical patients (van Loenhoud et al. 2019; Saczynski et al. 2014; Borchers et al. 2024). Since the interventions aiming at cognitive reserve enhancement, such as participation in educational programs or engaging in leisure activities, are shown to have positive effects on cognitive function in older adults (Lenehan et al. 2016), preoperative cognitive training or prehabilitation for cognitive reserve enhancement has been considered as a delirium preventive strategy in the patients undergoing noncardiac and cardiac surgeries (Humeidan et al. 2021; Jiang et al. 2024). However, the role of brain microstructural changes in preoperative cognitive reserve and subsequent POD development has not been addressed.

## 5 Brain Microstructural Alterations and POD

A previous meta‐analysis has indicated the MRI biomarkers of neurodegenerative and neurovascular changes associated with POD and PND (Kant et al. 2017). Recently, Kant et al. classified older patients scheduled for surgery into six brain phenotypes via MRI hierarchical clustering analysis, including three groups with limited burden brain pathology, one group with mainly atrophy, one group with mainly atrophy and SVD, and one group with multi‐burden brain pathology. Further correlation analysis showed 3.8 fold increase in POD risk in the multi‐burden group (Kant et al. 2021), shedding light on the underlying brain structural mechanism of POD. However, this classification of brain phenotypes is mostly based on MRI macrostructural features.

### 5.1 MRI Findings in Microstructural Alterations in POD Patients

Several clinical studies also investigated the microstructural changes linking to POD or PND. Although limited numbers of studies, they provide a landscape on microstructural features based on MRI metrics in POD patients. For example, low fractional anisotropy (FA) and mean kurtosis (MK) and, conversely, high mean diffusivity (MD) and FW, indicate microstructural abnormality. Preoperative DTI study demonstrated that microstructural abnormalities in the cerebellum, cingulum, corpus callosum, internal capsule, thalamus, basal forebrain, occipital, parietal and temporal lobes, and hippocampus were associated with POD incidence and severity, and after further adjustment with preoperative cognitive performance, microstructural alterations in the cerebellum, hippocampus, thalamus, and basal forebrain still remained associated with POD incidence and severity (Cavallari et al. 2016). While Fislage et al. (2022) study using preoperative diffusion kurtosis imaging (DKI) showed that presurgical microstructural abnormalities reflected by high MD in thalamus and thalamic nuclei were significantly associated with POD. Further, they analyzed DKI metrics in whole brain and found preoperative global MD was higher, while global MK and FW were lower in elderly patients with POD (Fislage et al. 2024). These evidences strongly indicate preoperative brain microstructural alterations or structural disconnectivity predispose elderly patients undergoing major surgery to POD. On the other hand, surgery and POD could also influence brain structure. Kant et al. (2023) found gray matter volume had a greater decrease 3 months after surgery when compared with nonsurgical controls, while compared with non‐POD, those patients with POD had greater decrease in preoperative to postoperative volume of gray matter but not white matter. And postoperative DKI study also demonstrated that postoperative global MD was lower after 3 months and higher after 1 year in patients with POD, suggesting POD may also induce a depletion of white matter. Further, the patients experiencing POD had long‐term changes in the periventricular, frontal, and temporal white matter (Cavallari et al. 2017). As a result, these microstructural abnormalities were also associated with cognitive deterioration across 1 year, which underlies trajectory evolution of neurocognitive disorders. These findings suggest the brain structural alterations not only predispose to but also result from POD. However, the same study group also indicated pre‐existing cerebral white‐matter hyperintensities, global brain atrophy, and hippocampal atrophy were not significantly associated with the incidence or severity of POD in older patients without dementia (Cavallari et al. 2015). Brain mineralization associated with iron deposition and calcifications, has been suggested as a risk factor of several neurodegenerative diseases. In a small‐size clinical study, the researchers investigated the role of preoperative brain mineralization in POD development in cognitively healthy elderly patients. They observed that among studied brain regions with susceptibility‐weighted MRI, only mineralization‐related hypointensity in posterior basal forebrain cholinergic system was associated with POD (Lammers‐Lietz et al. 2024), suggesting changes in microstructural components may also mediate POD occurrence. Nevertheless, given its exploratory nature, a large‐sample study is warranted to further confirm this finding. Interestingly, in certain specific surgical procedures, brain microstructural damage can even be improved postoperatively, for example, bariatric surgery can promote white and gray matter recovery (Michaud et al. 2020; Tuulari et al. 2016), reflecting that metabolic improvements induce brain microstructural and cognitive improvements.

### 5.2 Limitations of Current Studies

Several limitations in these studies should be noted. For example, (1) only macrostructural rather than microstructural changes is investigated in some studies; (2) the association between proinflammatory cytokines levels and microstructural disruption has not studied in surgical patients with POD; (3) the simple structural MRI scan is used, or dMRI just for visual inspection only. Application of multimodal microstructural neuroimaging techniques that detect neuroplasticity and even small volume shifts between the compartments (intra‐axonal, extra‐axonal, and FW/CSF) in white matter (Tardif et al. 2016; Karat et al. 2023), may be a promising approach to overcome this limitation.

### 5.3 Direction of Future Research

To further fill in the knowledge gap in microstructural alternations associated with POD, in future clinical studies, we should define (1) preoperative or postoperative brain microstructural changes associated with POD; (2) the most relevant microstructural changes structurally and spatially with POD; (3) the causal relationship among microstructural changes, neuroinflammation, and POD; and (4) the optimal neuroimaging techniques and metrics used to measure perioperative microstructural changes. These studies could provide structural risk factors for predicting POD, help to understand pathogenesis of POD, and establish the standard method to find specific structural biomarkers of POD.

## 6 Preoperative Brain Microstructure Evaluation

### 6.1 Neuroimage Techniques for Brain Microstructure Assessments

Numerous studies have investigated the characteristics of aging brain microstructure by using advanced neuroimaging techniques with gray matter (cortical thickness, surface area, and volume) and white matter (FA; mean, axial, and radial diffusivities) structural metrics. The changes in neural and nonneural dependent activity (e.g., synaptogenesis and changes in dendritic spine morphology) and changes in white matter (e.g., variation of axon diameter, myelin, packing density, and fiber geometry) contribute to the observed alterations in neuroimaging data. Each neuroimaging technique offers unique insights into the structural and compositional changes (Hagiwara et al. 2025). Structural MRI has played a pivotal role in the monitoring and understanding the healthy aging process, for example, by detecting macrostructural patterns of brain atrophy and microstructural lesions of gray/white matter. However, single‐contrast structural MRI is suboptimal for measuring microstructural changes, such as myelin water fraction and g‐ratio mapping. Other MRI‐based techniques, such as dMRI and magnetization transfer imaging, sensitive to the direction of water diffusion or myelin content, are increasingly being applied to study the brain microstructural changes during aging processes although both of them lack specificity. The most widely used DTI is a noninvasive water diffusion technique. Main metrics from DTI sensitive to microstructural changes are FA and MD. FA in white matter pathways is used to reflect directional coherence of fibers and MD in gray matter reflects magnitude of water molecule diffusion. Age‐related changes in DTI have been found in various white matter regions, while the greatest change commonly found in the anterior corpus callosum. These changes are frequently attributed to deficits in myelin integrity. The DKI also measures non‐Gaussian diffusion and may provide complementary information to DTI. And MK, a measure of tissue complexity, is found to increase in white matter during maturation and decrease in healthy aging. Another advanced dMRI analysis technique, neurite orientation dispersion and density imaging, can quantify the density and dispersion of neurites (i.e., axons and dendrites), however, it can be also used to investigate neuropathological changes in the brain. These neuroimaging measurements can not only track the longitudinal microstructural changes associated cognitive decline, but also necessitate differentiation between normal aging and neurodegeneration. Therefore, they could be also used for assessment of preoperative brain structure including brain volume, cortical thickness, and integrity of gray/white matter in elderly patients, and importantly, to find resilient or susceptible microstructural biomarkers to identify those at high risk of POD.

### 6.2 Other Methods for Brain Microstructure Detection

Beside whole‐brain structural neuroimaging analyses, other indirect methods can also be used for predicting brain microstructural changes. Since retinal thinning and microstructural changes have specific associations with brain atrophy primarily involved in vision and cognition, such as lower hippocampal volume and lower thickness of the occipital region (Jiang et al. 2025; Liu et al. 2016), we speculate that retinal microstructural changes may be a useful substitute for brain microstructural changes linked to cognitive impairments.

Another biomarker DNA methylation age of peripheral blood cells is also strongly associated with cortical thinning and white matter disease burden (Bonham et al. 2024). This epigenetic age may represent a promising predictor for distinguishing cognitively normal aging across MCI to AD.

## 7 Potential Interventions for Brain Microstructural Improvements

### 7.1 Cognitive Training Targeting Microstructure

The cognitive interventions to effectively prevent age‐associated brain impairments have received substantial attentions. The animal studies have demonstrated that cognitive or memory training could improve deficits in spatial memory and attention after TBI through modulating microstructural organization in the hippocampus and cingulum (Braeckman et al. 2020). The clinical studies also reveal the effects of cognitive training on brain microstructural modulation. The study has indicated the white matter microstructural changes in response to a memory training both in young and older adults, suggesting that microstructural plasticity is preserved into older age (de Lange et al. 2017). The involved brain areas include corpus callosum, cortico‐spinal tract, cingulum bundle, superior longitudinal fasciculus, and anterior thalamic radiation. This cognitive training‐induced microstructural improvements is related to memory improvement. And a simple 4‐week verbal articulation training intervention significantly increases gray matter volume and improves white matter integrity in elderly adults as evidenced by increased density of white matter tracts in certain brain regions (Hsu et al. 2024). Moreover, training on a perceptual decision task alters subcortical (pulvinar and hippocampus) myelination and its functional connectivity to visual cortex and relates to decreased visual cortex GABAergic inhibition (Ziminski et al. 2023).

### 7.2 Brain Stimulation Targeting Microstructure

Recent studies also suggest that the combination of behavioral training and concurrent brain stimulation techniques, transcranial direct current (tDCS) or magnetic stimulation (TMS), may induce improvements in white/gray matter microstructure, neural plasticity, and cognitive benefits in advanced age (Polanía et al. 2018; Antonenko et al. 2023). These evidences suggest a link among cognitive interventions, microstructural modulations, and cognitive improvements. Since microstructural alterations is part of a neurobiological substrate for neurobehavioral improvements in older adults (McPhee et al. 2019), receiving preoperative cognitive training or brain stimulation may be a preventive strategy against POD through influencing microstructural plasticity. Two recent studies by same study team have investigated the efficacy of brain stimulation in preventing POD. In first one, adult patients receiving two preoperative sessions of active tDCS for anxiety showed less incidences of both perioperative anxiety and POD than those receiving sham tDCS (Li et al. 2024). In another study, one preoperative session of active tDCS over the left dorsolateral prefrontal cortex decreased the incidence of POD in elderly surgical patients (Tao et al. 2023). Whether POD‐reducing effect by preoperative tDCS attributes to its effects on microstructural plasticity in these surgical patients needs to further study.

### 7.3 Physical Training and Microstructural Alterations

Compared with cognitive training, the beneficial effects of physical training on age‐related microstructural deterioration is uncertain. It is reported that 6‐month fitness training significantly increases hippocampal volumes and improves hippocampal microstructure in healthy participants aged 59–74 years with a sedentary lifestyle (Kleemeyer et al. 2016), while Callow et al. (2021) even found a single 30‐min moderate to vigorous aerobic exercise session could lead to hippocampal neuroplasticity and microstructural alterations associated with cognitive improvements in healthy older adults. In contrast, a 12‐week aerobic training program fails to change cognitive function or brain structure in older adults (Sexton et al. 2020). In animal studies, physical activity has shown to improve PND (Böckmann et al. 2023; Oroszi et al. 2022), however, adhering to an exercise regime is challenging for certain older patients due to the physical weakness and possible limb dysfunction, these controversial evidences further make physical exercise not a suitable preoperative option to improve cognitive reserve and brain microstructure.

### 7.4 Duration of Cognitive Training and Microstructural Modulation

The duration of cognitive training could affect the modulation of brain microstructure. The macroscopic changes are found mainly after weeks to several months of training in humans, however, the growing evidence has shown that modifications to axons and myelin occur not only as a result of long‐term learning, but also after short training periods. For example, imaging of motor cortical networks revealed a rapid and specific microstructural alteration after a few hours of training in animals (Taubert et al. 2016), and a continuous visuomotor sequence leaning for five consecutive days in humans could successfully improve behavioral performances associated with significant changes in white matter microstructure and functional connectivity (Tremblay et al. 2021). Interventions aiming at enhancement of cognitive reserve, such as participation in cognitive programs or engaging in leisure activities, have been shown to exert positive effects on cognitive function in older adults, however, the optimal strategy to promote cognitive reserve against POD is not established for elderly surgical patients (Lau et al. 2024). In the future, computer‐assisted cognitive training based on structured learning may facilitate the surgical patients to complete such an intervention program within a preoperative time‐restricted framework. Furthermore, it is reported that the training‐related microstructural improvements subside over time (de Lange et al. 2018), therefore continuous cognitive training following surgery may be a premise for the enduring attenuation of POD incidence and severity.

## 8 Perspectives

Recent clinical studies have investigated the roles of perioperative managements in POD occurrence. In cardiac and noncardiac surgeries, EEG‐guided depth of anesthesia seems not a key factor influencing the development of POD (Wildes et al. 2019; Deschamps et al. 2024), while in major surgery including cardiac surgery, a lighter anesthesia (BIS = 50) led to lower incidence of POD (Evered et al. 2021). Meanwhile, the used anesthetic agents (propofol vs. sevoflurane) and anesthetic types (regional anesthesia vs. general anesthesia) during the surgical procedures may be also not the determinants of POD development (Foley and Djaiani 2025; Mei et al. 2020; Li et al. 2022), however, some studies provided contrary results (Cao et al. 2023; Fanelli et al. 2022). A large‐size (2352 elderly patients) retrospective cohort study even suggested that intraoperative hypotension (defined as mean arterial pressure < 65 mmHg) is not associated with POD in elderly patients undergoing elective noncardiac surgery (Zarour et al. 2024), while a larger (including 316,717 adult patients) retrospective study indicated a mean arterial pressure < 55 mm Hg duration‐dependently increased POD risk in a similar surgical population (Wachtendorf et al. 2022). Moreover, Hughes et al. (2017) indicated that cognitive impairment after major noncardiac surgery and critical illness was not associated with the surgery and anesthesia exposure but is predicted by baseline education level and in‐hospital delirium. These controversial data suggest perioperative management may play an important role in preventing POD occurrence, while those patients at‐risk of POD may also have inherent preclinical brain abnormalities.

The integrity and plastic potential of brain microstructure are crucial for optimal brain function, while structural damage could adversely affect functional brain networks and thus cognitive function. Therefore, we reasonably speculate here that as a predisposing factor, pre‐existing vulnerability in specific brain microstructure is linked to POD development in elderly patients, while surgery‐triggered neuroinflammation may further aggravate brain microstructural damage (Yang et al. 2024). This “double‐hit” pattern may finally lead to POD and even PND. Therefore, understanding the heterogeneous brain microstructural alterations and discriminating the patients with accelerated brain aging (vulnerable brain) from those with resilient brain aging (healthy brain) may be critical to find the specific microstructural signatures associated with POD. Namely, identifying those at high risk of POD linked to vulnerability to surgery/anesthesia would be helpful to establish targeted interventions during perioperative period. It is also essential for developing strategies aimed at improving brain resilience including promotion of microstructural integrity and resolution of inflammation, which can prevent POD occurrence.

## Author Contributions

**Yali Chen**: methodology, data curation, funding acquisition, writing–original draft. **Huanyu Luo**: investigation, writing–original draft. **Jing Dong**: methodology, validation, writing–review and editing. **Jun Zhang**: conceptualization, methodology, data curation, funding acquisition, writing–review and editing.

## Ethics Statement

The authors have nothing to report.

## Consent

All authors agree to publish this manuscript in this journal.

## Conflicts of Interest

The authors declare no conflicts of interest.

## Peer Review

The peer review history for this article is available at https://publons.com/publon/10.1002/brb3.70872

## Data Availability

The data that support the findings of this study are available on request from the corresponding author. The data are not publicly available due to privacy or ethical restrictions.
